# Relevance of Surface Neuronal Protein Autoantibodies as Biomarkers in Seizure-Associated Disorders

**DOI:** 10.3390/ijms20184529

**Published:** 2019-09-13

**Authors:** Gabriela Dumitrita Stanciu, Veronica Bild, Daniela Carmen Ababei, Razvan Nicolae Rusu, Sorin Ioan Beschea Chiriac, Elena Rezuş, Andrei Luca

**Affiliations:** 1 Grigore T. Popa University of Medicine and Pharmacy, Center for Advanced Research and Development in Experimental Medicine (CEMEX), 16 Universității street, Iasi 700115, Romania; gabriela-dumitrita.s@umfiasi.ro (G.D.S.); andrei.g.luca@umfiasi.ro (A.L.); 2 Grigore T. Popa University of Medicine and Pharmacy, Pharmacodynamics and Clinical Pharmacy Department, 16 Universității street, Iasi 700115, Romania; dana.ababei@gmail.com (D.C.A.); razvan.nicolae.rusu@gmail.com (R.N.R.); 3 University of Agricultural Sciences and Veterinary Medicine “Ion Ionescu de la Brad”, Faculty of Veterinary Medicine, 8 M. Sadoveanu Alley, Iasi 700489, Romania; 4 Grigore T. Popa University of Medicine and Pharmacy, Department of Rheumatology and Physiotherapy, 16 Universității Street, Iasi 700115, Romania; elena.rezus@umfiasi.ro; 5 Grigore T. Popa University of Medicine and Pharmacy, Pneumology Department, 16 Universității Street, Iasi 700115, Romania

**Keywords:** autoantibodies, neuronal surface proteins, seizure disorders, biomarkers, diagnostic, immunomodulatory therapy

## Abstract

The detection of neuronal surface protein autoantibody-related disorders has contributed to several changes in our understanding of central nervous system autoimmunity. The clinical presentation of these disorders may be associated (or not) with tumors, and often patients develop an inexplicable onset of epilepsy, catatonic or autistic features, or memory and cognitive dysfunctions. The autoantigens in such cases have critical roles in synaptic transmission and plasticity, memory function, and process learning. For months, patients with such antibodies may be comatose or encephalopathic and yet completely recover with palliative care and immunotherapies. This paper reviews several targets of neuronal antibodies as biomarkers in seizure disorders, focusing mainly on autoantibodies, which target the extracellular domains of membrane proteins, namely leucine-rich glioma-inactivated-1 (LGI1), contactin-associated protein-like 2 (CASPR2), the *N*-methyl-D-aspartate receptor (NMDAR), γ-aminobutyric acid receptor-B (GABA_B_R), the glycine receptor (GlyR), and a-amino-3-hydroxy-5-methyl-4-isoxazolepropionic acid receptors (AMPARs). In order to restore health status, limit hospitalization, and optimize results, testing these antibodies should be done locally, using internationally certified procedures for a precise and rapid diagnosis, with the possibility of initiating therapy as soon as possible.

## 1. Introduction

Over the last decade, contrary to established knowledge, a series of autoantibody-mediated seizure-associated disorders have been identified. This fact is beginning to overturn previous theories that regarded the brain as immune-privileged and protected by an impermeable blood–brain barrier [1]. 

The clinical picture of nervous system disorders related to autoantibodies against the various neuronal surface proteins is rapidly increasing, and novel associated conditions have been presented in recent years. In human medicine, most of these patterns are reversible through immunomodulatory treatments, and it is known that an early diagnosis with the discovery of these autoantibodies is essential for an early therapeutic approach. In consequence, some of these autoantibodies have begun to be part of the evaluation for patients with an inexplicable onset of epilepsy or memory and cognitive dysfunctions [2]. Currently there is limited knowledge on how autoantibodies against the different neuronal surface proteins could determine such a variety of diverse clinical patterns, and the identification of new autoantibodies is one of the state-of-art methods used in elucidating the pathogenesis and classification of these seizure-associated disorders.

The accelerated expansion of studies over antibody-mediated seizure syndromes has made it possible to identify and cure disorders that would otherwise have been less defined. Without a doubt, the development and implementation of animal model autoantibodies against various neuronal surface proteins represents a future direction in elucidating how these types of antibodies enter into the nervous system. Moreover, antibody binding, internalization, and damage of the specific surface antigens, together with the activation of complements, are designated factors involved in physio-pathological mechanisms [3]. At this point, there is a demonstrated need to extend the knowledge on the pathophysiology of these conditions and to improve diagnostic methods, which will subsequently have obvious positive consequences on more effective and precise therapies.

In this paper, we sought to highlight the relevance of autoantibodies against surface neuronal proteins as biomarkers in seizure-related disorders and to highlight that identifying positive extracellular antineural autoantibodies in clinical practice should be encouraged for better clinical management.

Addressing seizure-related disorders associated with antibodies against cell-surface antigens was chosen because they differ from those related to intracellular antigens regarding a number of main aspects, such as that cell-surface target antigens are disrupted by the antibodies, the link with malignancy is much less consistent, and symptoms are present that can more commonly be reversed with treatment. In addition, the symptoms relate mainly to the disruption of the target antigen.

## 2. General Features of Autoantibodies against Surface Neuronal Proteins

**Figure 1:**
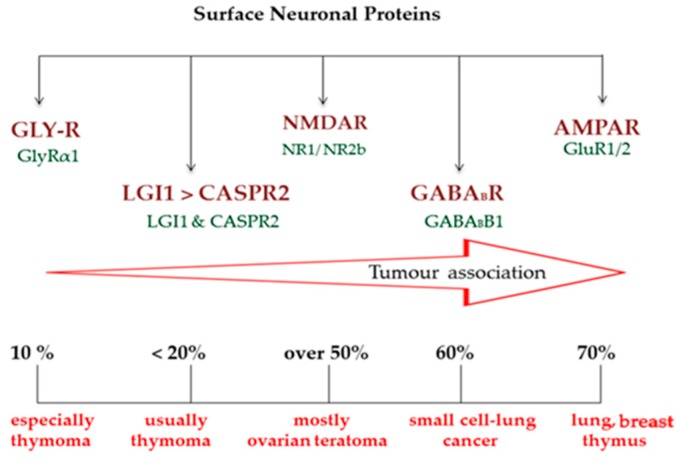
Schematic diagram of specific antibodies against surface neuronal proteins (leucine-rich glioma-inactivated-1 (LGI1), contactin-associated protein-like 2 (CASPR2), the *N*-methyl-D-aspartate receptor (NMDAR), γ-aminobutyric acid receptor-B (GABA_B_-R), the glycine receptor (GlyR), and a-amino-3-hydroxy-5-methyl-4-isoxazolepropionic acid receptors (AMPARs)) associated with seizure disorders. The associated autoantibodies are distributed into pathogenic entities that target extracellular neuronal areas of LGI1 and CASPR2, the NR1 and NR2b subunits of the NMDARs, the GABA_B_R1 subunit of the GABA receptors, the GluR1/2 subunits of the AMPAR (from which 70% of cases with increased AMPAR are of paraneoplastic origin), and the GlyRα1 subunit of the GlyRs.

Recently, cases of seizure disorders in which antibodies that target extracellular domains of cells or synaptic proteins, including leucine-rich glioma-inactivated-1 (LGI1), contactin-associated protein-like 2 (CASPR2), the *N*-methyl-D-aspartate receptor (NMDAR), γ-aminobutyric acid receptor-B (GABA_B_R), the glycine receptor (GlyR), and a-amino-3-hydroxy-5-methyl-4-isoxazolepropionic acid receptors (AMPARs), are recognized as having increased frequency (Figure 1) [4,5,6,7,8].

Although the idea that the immune system is involved in the pathology of AE is widely accepted, some researchers are not convinced. At this point, the role of autoantibodies or the contribution of B and T cells are yet to be determined, and thus the physiopathology of this disease still requires further studies [9].

### 2.1. LGI1 and CASPR2 

It is now recognized that voltage-gated potassium channel (VGKC) antibodies target many proteins. Known as the VGKC complex, it is detected in patients with peripheral nerve hyperexcitability, encephalitis, Morvan syndrome, or various other disorders. Initial research has suggested that patients’ antibodies bind to the VGKCs Kv1.1 and Kv1.2. [10,11]. Interestingly, it has currently come to light that leucine-rich glioma-inactivated-1 (LGI1) and contactin-associated protein-like 2 (CASPR2) have been identified as typical target proteins in most of the pathologies involved, which was previously suggested to be related to antibodies against the voltage-gated potassium channel (VGKC) complex [4,12].

LGI1 describes a secreted glycoprotein that binds to presynaptic proteins ADAM1 and ADAM23 and postsynaptic ADAM22, with a significant role in synaptic transmission by regulating the impairment of the AMPAR function, as well as the Kv1.1 and Kv1.2 subunits. This trans-synaptic tuning is considered to display an antiepileptic outcome [4]. 

CASPR2 represents a transmembrane protein located juxtaparanodally to myelinated axons, which at this level, together with contactin-2 and protein 4.1B, may concentrate Kv1.1 and Kv1.2 channels [4]. Its extracellular area interconnects with contactin-2, and via protein 4.1B it can connect to the cytoskeleton [6]. Existing data offer two possible pathogenic pathways involving LGI1 antibodies, such as the reduction of LGI1–ADAM complex formation and the alteration of the dendritic density of the neurons situated in the thalamus and hippocampus, whereas CASPR2 antibodies most likely act by disturbing axonal potassium currents [1,4,5,7,13].

### 2.2. GlyR 

An ionotropic receptor consisting of five distinct subunits (α1-4 and β), GlyR is found in the brainstem and medulla, and its activation produces a chloride flux that has an inhibitory role in the majority of mature neurons that possess a low intracellular chloride concentration [14]. In humans, hereditary hyperekplexia is the consequence of genetic destruction of GlyR subunits, and similar conditions have been observed in mice, dogs, and cattle [15]. However, its pathogenicity in seizures disorders is not well defined, and data on long-term follow-up of patients with epilepsy containing GlyR-Ab do not exist [16].

### 2.3. NMDAR 

The *N*-methyl-D-aspartate receptor, also recognized as the NMDA or NMDAR receptor, is one of the postsynaptic glutamate receptors found in neuronal cells, with a crucial role in neural plasticity, learning, and memory functions [17]. Antibodies against the extracellular NR1 and NR2b subunits of the NMDAR are related to limbic encephalitis (LE), epilepsy, and systemic lupus erythematosus, with patients presenting symptoms such as seizures, autonomic disturbance, amnesia, loss of memory, and psychiatric signs [18,19]. The mechanisms that support the pathogenicity of NMDAR antibodies include antagonizing or agonizing the NMDAR, which leads to receptor internalization and degradation, producing a reduction in receptor function or interesting complement-mediated neuronal impairments [20,21,22].

### 2.4. GABA_B_R 

The γ-aminobutyric acid receptor-B (GABA_B_R) describes a G protein-coupled receptor capable of mediating both presynaptic and postsynaptic inhibition. Each receptor consists of two subunits: GABA_B_1, which has an essential role in agonist binding, and GABA_B_2, which is required to perform intracellular signaling. GABA_B_Rs are predominantly situated in the presynaptic and postsynaptic areas of the hippocampus, thalamus, and cerebellum [23]. The antibodies bind only to the extracellular domain of the GABA_B_1 subunit, causing a selective disruption of synaptic signaling, although they do not appear to facilitate antibody-mediated crosslinking and internalization. A direct blockade of GABA_B_R leads to influencing the occurrence of seizures and cognitive deficits. The primary mechanisms through which GABA_B_R antibodies have an effect on patients are still under study [15,23]. 

### 2.5. AMPAR

One of the transmembrane ionotropic receptors of the glutamate receptor family, the alpha-amino-3-hydroxy-5-methyl-4-isoxazolepropionic acid receptor (AMPAR) may mediate in the brain, mostly in fast, excitatory neurotransmissions, with an essential role in synaptic plasticity, memory function, and process learning [24]. Structurally, these receptors are found as heterotetramers designed from combinations of the GluA1-4 subunits (previously named GluR1 or GluR2). Antibodies to the extracellular domain of the GluA1 and GluA2 subunits of the AMPAR are associated with conditions such as limbic encephalitis, epilepsy, or ataxia. The mechanisms used by these antibodies to constrain receptor function appear to be similar to those of NMDAR antibodies, initiating a reversible reduction in synaptic AMPARs and linked synaptic flows [5,15,25]. 

## 3. Seizure Disorders Associated with Surface Neuronal Protein Autoantibodies

**Figure 2:**
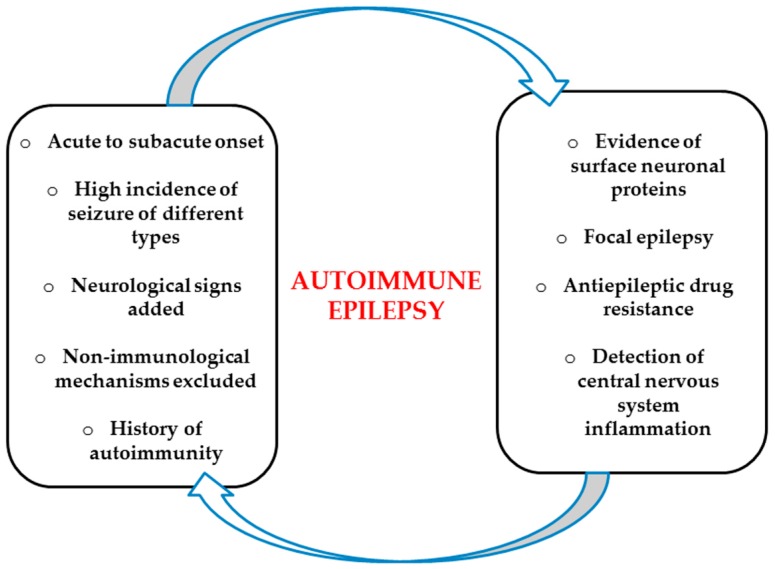
Clinical factors and features in the diagnosis of autoimmune epilepsy syndromes. In most cases, autoimmune epilepsy has an acute or subacute debut, as opposed to a progressive clinical evolution. Frequently, a history of additional autoimmune disorders can be present. Patients with this condition may concurrently express cognitive deficiency, encephalopathy, important psychiatric and behavioral alteration, signs of movement disorder, and novel atypical headaches even before the seizures. The incidence of seizure rates is significantly higher in cases of autoimmune epilepsy than in epilepsy with other origins, often with a frequency of several times per day. The seizures usually have characteristics that indicate the contribution of limbic regions. Manifestations in accordance with FBDS should quickly lead to autoantibodies against surface neuronal protein testing.

Epilepsy is estimated to affect more than 65 million people globally, up to 30% of whom continue to be inadequately managed despite available treatment [26]. Identifying a curative etiology may be the only way to achieve an important seizure reduction in the targeted patient population. In the same direction, finding an autoimmune etiology with therapeutically intractable epilepsy offers the opportunity of immunomodulatory therapy, which would not normally be considered. The main clinical factors and features that would suggest the probability of autoimmune epilepsy are summarized in Figure 2.

Most encephalitis cases considered to be past idiopathic are known to be immune-mediated. These conditions mainly touch the structures of the limbic system, including the temporal medial lobes, the amygdala, the hippocampus, and the orbitofrontal cortex [27,28,29]. As a consequence, patients develop alterations in short-term memory and behavioral abnormalities often associated with seizures and limbic system signal modifications in the imaging analysis, with different degrees of cerebrospinal fluid inflammation and antibodies against neuronal surface proteins [28,29]. Until recently, almost all cases of limbic encephalitis *(*LE) were considered to be of paraneoplastic origin and mediated by immune responses against intracellular antigens, with a poor prognosis and minimal response to treatment [28,30]. Recent studies, however, have revealed that in an appreciable category of associated disorders, the antigens are on cell surface proteins [30,31].

Autoimmune encephalitis (AE) (with antibodies against neuronal cell surface antigens) is a pathology that, like many others, currently includes evolving concepts, and since many of them have overlapping features with LE, such as possible seizures, irritability, or hallucinations, at times the term LE is misused in describing some type of AE. For AE identification in the absence of LE manifestations, acute onset and increased seizure frequency, a history of autoimmunity or neoplasia, and conventional treatment resistance might lead to an early diagnosis [32].

To date, research has focused on various neuronal surface protein antibodies associated with subacute autoimmune seizure disorders. Nevertheless, syndromes have developed that may extend the scope of these conditions beyond autoimmune epilepsies and encephalitis. Lately, antibodies against the neuronal cell surface adhesion molecule IgLON5, which had an unknown function, were identified in eight patients with a progressive neurodegenerative abnormal sleep movement disorder. The average age of these patients was 59 years, and five of them were women. None of the cases had cerebrospinal fluid (CSF) anomalies, MRI FLAIR hyperintensities, atypical electroencephalograms (EEGs), or tumors. The disease was ultimately fatal, and immunotherapy had no response. The autopsy exposed in two patients a new tauopathy, including the brainstem and hypothalamus. This finding underlines that autoimmune mechanisms may cause some chronic neurodegenerative syndromes, while it remains to be determined whether anti-IgLON5 antibodies are pathogenic or rather a biomarker of this neurodegenerative process [33]. 

Other antibodies to neuronal surface proteins or synaptic receptors have been recognized in nonencephalitic syndromes. For example, antibodies against the dopamine 2 receptor have been identified in some cases of Tourette syndrome, Sydenham chorea, and in a small subcategory of children with basal ganglia encephalitis [34]. Moreover, the spectrum of signs and conditions linked to VGKC complex antibodies other than LGI1 and CASPR2 is growing and comprises nonimmune-mediated CNS disorders, such as hepatic encephalopathy [35]. The relationship of these antibodies to the associated syndromes is not yet fully known.

### 3.1. LGI1 and CASPR2 Autoantibodies

LGI1 antibodies are involved in limbic encephalitis (over 90%) [36,37,38], but a small part of patients may develop epilepsy [39,40,41], a subacute encephalopathy, or Morvan syndrome [42,43]. The rich expression of these autoantibodies in the hippocampus and the limbic system guarantees the vulnerability of these epileptogenic regions [44]. With an annual increasing incidence rate (0.63–0.83/million), LE with LG1 antibodies has an onset of age estimated between 61 and 64 years, where 55%–66% of the patient population is men [38,45,46,47,48,49,50]. LE with LGI1 antibodies, which is commonly encountered in the lack of a related neoplasm, has primary symptoms in most patients, such as cognitive impairments, seizures, psychiatric and behavioral conditions, sleep abnormalities, and autonomic disturbances [38,44,47,51]. This condition usually reaches maximum severity within six months, during which patients may develop memory and behavior impairments, often accompanied by spatial disorientation and seizures in 90% of cases [43,51]. Facio-brachial dystonic seizures (FBDSes) are specific to LGI1 encephalitis, and they appear in up to 70% of patients: they have a duration of less than 30 s, they have a frequency of 10–100 times per day, and they most often occur before the development of cognitive disturbance [38,43,44,51]. However, in the course of the disease, tonic-clonic seizures are recorded in the severe stage or subtle focal seizures may occur in two-thirds of patients, although they are more difficult to highlight because patient descriptions are frequently vague [45,52,53]. Found through neuroimaging, LE with LGI1 antibodies is characterized by enlargement, T2 hyperintensity, and limited diffusion or contrast enhancement of limbic structures and evidence of inflammation in the cerebrospinal fluid [40,54]. Electroencephalograms (EEGs) highlight focal slowing or epileptic discharges in more than half of patients. Remarkably, only longer FBDSes may correlate with electrodecremental events [43,55,56], while the majority of EEGs display interictal epileptiform discharges in the course of dyscognitive or autonomic focal seizures. Tumors of various types are found in nearly 11% of patients [55]. 

Contrary to the LGI1 antibodies that are present in definite conditions, CASPR2 antibodies appear in combination with widespread clinical trial syndromes. The majority of these disorders display a large overlap of signs, reflecting the repeated implication of both the central and peripheral nervous system. Moreover, the clinical spectrum is limited, as this condition is quite rare. CASPR2-antibody-mediated syndromes have a robust male prevalence (84–88%), for which studies have not yet found solid justifications. The mean age of onset is approximately 60–70 years, with lower averages for women [45,57], with nearly 200 reported cases so far [43,57,58,59]. Most people with CASPR2 antibodies present with LE or Morvan syndrome [45,46]. The symptoms may be subtle and often difficult to mention by patients or family members, but even so, seven core types have been identified: cerebral and cerebellar symptoms, autonomic dysfunction, insomnia, peripheral nerve hyperexcitability, neuropathic pain, and weight loss [45,46,60,61]. Seizures are present in half of patients, with 80% of them developing cognitive impairments. The spontaneous muscular activity registered in over 50% of cases with this condition translates to myokymia, fasciculation, and muscle cramps. The evolution of the disease is often for several months, but in ~ 30% of cases, the progression extends over a one-year period [46]. Regular laboratory analyses are frequently normal. Slightly raised cerebrospinal fluid (CSF) cell counts or proteins can be observed, but unremarkable CSF is detected in over 75% of patients. Seventy percent of patients may present with a normal brain MRI, but bilateral hyperintensity of the temporal lobe can be detected [21,46]. EEG recordings are nonspecific, tumors are encountered in 20% of cases, and usually thymoma [62] LGI1 and CASPR2 antibodies can be achieved with both serum and CSF analysis or with a combination of cell-based assays over brain tissue immunohistochemistry or immunocytochemistry with cultured live neurons [35,46,57,63]. The most common therapies involve steroids, intravenous immunoglobulin, or a combination of both, with a part of the patient population receiving second-line treatment. In a series of studies, the positive treatment response was from 79% to 90% [43,46,57,62,63]. 

**Figure 3:**
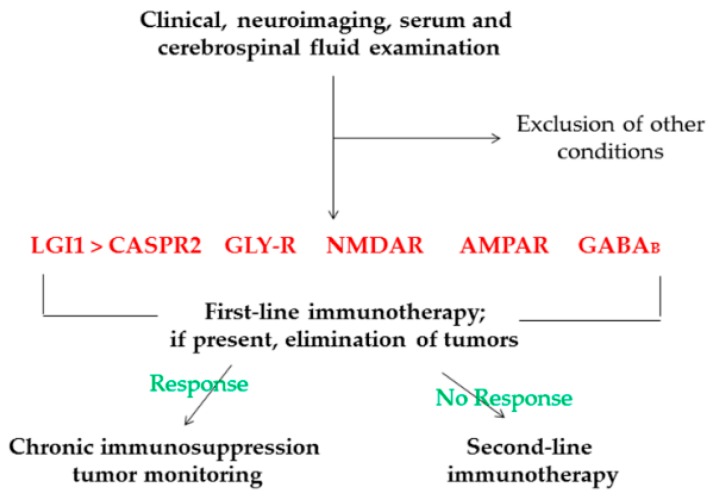
Diagnosis and treatment protocol after clinical, neuroimaging, serum, and cerebrospinal fluid examination with determination of specific antibodies and therapeutic decision with respect to response exerted after the first-line immunotherapy.

In light of similar clinical presentations, the diagnosis of LGI1 and CASPR2 antibody syndromes needs to be differentiated from other types of autoimmune or viral encephalitis, Hashimoto’s encephalopathy, and Creutzfeldt’s syndrome [64,65]. With corroborating clinical features with paraclinical findings, diagnoses of this kind of pathology are frequently correct and have good or very good responses to immunotherapy (Figure 3) [27,28,45,60,66]. However, these conditions are considered to still be underdiagnosed and therapeutically undermanaged [60,67,68]. More insight during clinical manifestations associated with neuroimaging data could be a means to improve and refine the diagnosis protocol of these diseases. 

### 3.2. GlyR Autoantibodies

To date, autoantibodies against the α1 subunit of GlyR have been mostly associated with stiff-person syndrome and progressive encephalomyelitis with rigidity and myoclonus, followed by fewer cases of LE and epilepsy, which are generally restricted to adulthood [14,69,70,71]. Summing up, in only a few dozen patients, GlyR autoantibodies were recorded in 3–6% of cases with epilepsy, with a more pronounced preference in patients with well-established epilepsy versus new-onset seizures and focal as opposed to generalized forms of epilepsy [72]. Regardless of their character in seizure pathogenesis, GlyR antibodies might be a biomarker of response to immunosuppressive therapies (Figure 3), mainly in patients with epilepsy resistant to antiepileptics and unusual clinical progressions. In addition to a well-established link between GlyR autoantibodies and entities such as stiff-person syndrome and progressive encephalomyelitis with rigidity and myoclonus (PERM) (presented in Table 1) [73], new studies have highlighted the possibility of epilepsy refraction with conventional therapy, but with good response to immune therapy (cases that present detectable GlyR autoantibodies) [74]. An extensive spectrum of neuroimagistic features upon presentation of frequently notable brainstem dysfunction, focal epileptic activity in EEG analysis, and slight MRI or CSF indication of inflammation were observed in single or three-patient series. Tumors, especially thymoma tumors (around 10%), are less commonly associated with these conditions [16,69,75,76].

### 3.3. NMDAR Autoantibodies

NMDAR encephalitis, a synaptic autoimmune condition in which autoantibodies target the NR1 or NR2b subunits of the NMDAR in the brain, causing their removal from synapses, is perhaps the most frequent antibody-associated encephalitis [91]. A high percentage of patients are young women or children who first develop subacute psychiatric disturbance, often followed by seizures, consciousness decline, autonomic dysfunction, movement disorders, and hypoventilation [19,81,82]. MRI data analysis is often not informative, with only pleocytosis at the onset of the disease being quite common [58,92]. EEG examinations have described diffuse slowing over ample numbers of patients. In comparison, the presence of extreme delta brushes, which have an incidence varying between 0% and 100%, represents a pathognomonic EEG pattern for NMDAR encephalitis [93,94,95]. In nonparaneoplastic patients, recurrences can occur in 20–25% of cases [58,92]. In NMDAR encephalitis, the CSF and/or serum analysis is an essential part of diagnosis, especially because other laboratory methods and imaging tests are not so pertinent [96,97]. According to some studies, NMDAR encephalitis antibody analysis in CSF is very sensitive and specific, with false positive or negative results recorded only when serum is tested alone [98]. NMDAR encephalitis therapy is still a challenge, as no complete guidelines have been published so far. Nevertheless, a substantial part of authors agree that the therapy must be directed both to the cause and to the consequences of the clinical spectrum of encephalitis (Figure 3) [97]. 

### 3.4. GABA_B_R Autoantibodies

GABA_B_R autoantibodies are typically associated with limbic encephalitis, describing an affinity toward prominent and severe seizures or status epilepticus. In more than 60% of patients, small cell lung cancer or neuroendocrine tumors are identified [21]. The encephalitis associated with GABA_B_-R antibodies may occur within very wide age limits, ranging from 24 to 75 years, with a median of 62 years, out of which around 50% of patients are women [99]. Seizures, either partial or generalized, are frequently difficult to manage, because they are refractory to various antiepileptic compounds and instead display a favorable response to immunotherapy [83]. In EEG evaluations, GABA_B_ receptor antibody patients frequently have focal/generalized temporal lobe epileptic activity, with or without universal slowing [21,84]. Memory impairments, personality alteration, and communication difficulties are characteristic signs of LE, while MRIs and positron emission tomography (PET) designate unilateral or bilateral medial temporal T2 high signals in the majority of patients [84,100]. Diagnosis needs the corroboration of typical findings (MRI, CSF, EEG, and/or histopathology), with the identification of GABA_B_R antibodies in serum and/or CSF [101]. In GABA_B_R LE, standard care is lacking, and the response varies widely. However, most researchers agree that the use of immunotherapies [99] and a mixed treatment is superior to monotherapy in reducing the degree of recurrence and increasing the rate of recovery (Figure 3) [40]. 

### 3.5. AMPAR Autoantibodies

With only a dozen cases diagnosed so far, AMPAR autoantibodies associated with encephalitis are often accompanied by an acute to subacute onset, with the signs being dependent on the area of the brain involved, such as the limbic and cerebral encephalitis or encephalomyelitis [85,87]. LE is more common in middle-aged women with symptoms of behavioral dysfunction and short-term memory damage concurrent with cognitive disturbance and seizures in almost 50% of cases. About 70% of patients are related to substantial malignancies comprising the lung, breast, or thymus [89,102]. The AMPAR encephalitis diagnosis should comprise the corroboration of anamnestic data, detailed examination, neuroimaging information, CSF analysis, EEG studies, and the exclusion of other possible sources. Most patients present abnormal MRI signals in the medial temporal lobes, with predominant lymphocytic pleocytosis and the occurrence of antibodies in the CSF evaluation [89,103,104]. Brain expression analysis reveals nonspecific anomalies such as focal or generalized attenuation, epileptiform patterns, and periodic lateralized epileptiform discharges. The AMPAR antibody condition usually responds to precise treatment of the tumor and immunotherapy but has a predisposition to relapse (Figure 3) [66]. 

This new area of immune-mediated seizure disorders is captivating but challenging at the same time. Moreover, these conditions that are immunotherapy-responsive require more intensive research and consequently may be well defined as neuronal surface antibody-associated syndromes. Therapies must be tailored individually. Figure 3 summarizes the treatment protocol most often followed after clinical, neuroimaging, serum, and cerebrospinal fluid examination. So far, there are no strategies regarding specific immunotherapies for the different types of neuronal surface protein-mediated disorders.

Currently, the basic principle applied in the treatment of these syndromes is immunosuppression. The most complete data have been derived from a retrospective investigation of 501 patients with anti-NMDA receptor encephalitis, for which therapy and long-term results were accessible [27]. These patients most often were treated with first-line agents such as corticosteroids, intravenous immunoglobulins, or plasmapheresis, individually or in combination, and rituximab and/or cyclophosphamide as a second-line therapy. In this report, more than 90% of patients received first-line treatments and tumor removal when needed, and 53% of them improved within 1 month: 57% of patients who did not respond to first-line therapies received second-line treatments, while 43% continued first-line treatment or discontinued therapy. Comparing the patients from the latter groups, those who received second-line agents performed better and had fewer relapses.

## 4. Surface Neuronal Proteins and Animals

In vivo preclinical models and human genetic mutations may offer valuable opportunities for understanding the molecular and clinical characteristics of surface neuronal protein autoimmunity. 

LGI1 is associated with both human and animal epilepsy, and it is a neuronal secreted protein not encoding a subunit of the ion channel [3,12,36,39,40,41,72]. Fukata et al. [4] have suggested an important role for LGI1 as an antiepileptogenic ligand. They found that LGI1 loss (LGI1^-/-^) in homozygote mice induces lethal generalized epilepsy, while in heterozygote ones (LGI1^+/-^), this leads to a decrease in seizure thresholds. Remarkably, in the brain, extracellular LGI1 simultaneously binds postsynaptic ADAM22 and presynaptic ADAM23, two epilepsy-associated syndrome receptors. In addition, it systematizes a trans-synaptic complex (containing presynaptic voltage-gated potassium channels and postsynaptic AMPA receptor scaffolds) that stabilizes synapses and increases neurotransmission. Similar results were reported by Chabrol et al. [105] in LGI1-null mice. Furthermore, Xie et al. [106] found that the effects of LGI1 on neuronal development are mainly reported in embryonic development and not in young mice. Consequently, LGI1 may be an essential cause of brain excitation, and the LGI1 gene-targeted mouse is a promising model for human epilepsy.

Encoded by the CNTNAP2 gene on chromosome 7, CASPR2 seems to be essential in neurodevelopment and in the precise placement of myelinated axons of the voltage-gated potassium channels in the central and peripheral nervous systems. CNTNAP2^-/-^ knockout mice demonstrated (before the onset of epileptic seizures) changes such as disturbances in neuronal migration, a decreased number of interneurons, atypical neural network activity, and autistic spectrum disorder deficits: these are features commonly found in pediatric patients with homozygous CNTNAP2 mutations [107]. 

Prosser et al. [108] have shown that GABA_B_ receptor-deficient murine models may display severe and prominent seizures and memory and learning disturbance, accompanied by behavior anomalies. These results suggest that GABA_B_ receptor antagonists could be valuable in psychiatric and neurological therapies. 

Surface neuronal protein-targeted rodents serve as a means of clarifying and establishing the pathogenesis and therapeutics of human seizure disorders.

## 5. Conclusions 

The clinical relevance of autoantibodies against surface neuronal protein positivity in seizure-associated disorders is sustained by favorable responsiveness to immunotherapy. Future research and therapeutic viewpoints will have essential value in determining aspects of drug-resistant seizure disorders and antibodies against surface neuronal proteins in order to recognize the patients who may benefit from immunotherapy. With the aim of reinstating health, limiting hospitalization, and optimizing results, testing these antibodies should be done locally using internationally certified procedures for a precise and rapid diagnosis, with the possibility of initiating therapy as soon as possible. Structured research oriented toward individually tailored therapies is needed in order to institute best practices for these patients.

## Figures and Tables

**Table 1:** Specific autoantibodies against surface neuronal proteins associated with seizure disorders.

Antibodies	LGI1 > CASPR2	GlyR	NMDAR	GABA_B_R	AMPAR
Syndrome	LE, epilepsy, and a subacute encephalopathy	Stiff-person syndrom, PERM, limbic encephalitis, cerebellar degeneration, or optic neuritis	Encephalitis	LE	LE and encephalitis
Main known target	LGI1 and CASPR2	GlyRα1	Mainly NR1 and NR2b subunits	GABA_B_R1	GluA1/2
Gender/number of cases	M ˃ F, more than 500 cases	M > F, dozens of patients	F ˃ M, ˃ 1000 patients	M > F, only dozens of patients	F > M, only dozens of cases
Clinical features and characteristic seizures	Cognitive impairments, seizures, psychiatric and behavioral conditions, sleep abnormalities and autonomic disturbances, three types of epileptic seizures: FBDS, CPS, GTC	Stiffness, rigidity, brainstem disturbance, cognitive involvement, rare but occasional seizures: GTC, CPS	Subacute psychiatric disturbance, consciousness decline, autonomic dysfunction, movement disorders, and hypoventilation, seizures: GTC, SE, CPS	LE with prominent seizures: CPS, GTC, SE	Early or prominent epileptic seizures: GTC, CPS
	EEG: focal or generalized slowing; CSF: ↑ cell count or unmatched oligoclonal bands, except LGI1; encephalitic lesions on MRI	Focal epileptic activity on EEG; encephalitic lesions on MRI and ↑ cell count	A pathognomonic EEG pattern, extreme delta brush; encephalitic lesions on MRI and ↑ cell count	EEG with focal/generalized epileptic activity; encephalitic lesions on MRI and ↑ cell count	EEG with focal epileptic activity; encephalitic lesions on MRI and ↑ cell count or unmatched oligoclonal bands
Favorable immune therapy response	Yes	Yes	Yes	Yes	Yes, relapses are common
Antibody screening	iIHC, RIA, CBA	CBA	iIHC, ELISA, CBA	iIHC, CBA	iIHC, CBA
References	[3,8,12,36,37,39,40,41,60,77]	[2,16,69,74,75,78]	[18,19,20,27,45,66,67,68,79,80,81,82]	[21,83,84,85,86]	[5,85,87,88,89,90]

Ab, antibody; AMPAR, a-amino-3-hydroxy-5-methyl-4-isoxazolepropionic acid receptor; CASPR2, contactin-associated protein 2; GABA_B_R, gamma-aminobutyric acid B receptors; GlyR, glycine receptor; LE, limbic encephalitis; LGI1, leucine-rich glioma-inactivated 1 protein; NMDAR, *N*-methyl-D-aspartate receptors; SE, status epilepticus; EEG, electroencephalogram; FBDS, facio-brachial dystonic seizure; GTC, generalized tonic-clonic; CPS, complex partial seizure; M, male; F, female; CSF, cerebrospinal fluid; MRI, magnetic resonance imaging; iIHC, indirect immunohistochemistry (immunofluorescence); RIA, radioimmunoprecipitation assay; CBA, cell-based assay; PERM, progressive encephalomyelitis with rigidity and myoclonus; ↑ cell count, an increased cell count

## Abbreviations

LGI1	Leucine-rich glioma-inactivated-1
CASPR2	Contactin-associated protein-like 2
NMDAR	*N*methyl-D-aspartate receptor
NR1 and NR2b	Subunits of the NMDAR
GABA_B_-R	γ-aminobutyric acid receptor-B
GluA1 and GluA2	Subunits of the AMPAR
GlyR	Glycine receptor
Ab	Antibody
AMPAR	a-amino-3-hydroxy-5-methyl-4-isoxazolepropionic acid receptors
LE	Limbic encephalitis
SE	Status epilepticus
EEG	Electroencephalogram
FBDS	Facio-brachial dystonic seizures
GTC	Generalized tonic-clonic
CPS	Complex partial seizure
M	Male
F	Female
CSF	Cerebrospinal fluid
MRI	Magnetic resonance imaging
PET	Positron emission tomography
iIHC	Indirect immunohistochemistry (immunofluorescence)
RIA	Radioimmunoprecipitation assay
CBA	Cell-based assay
